# The Timing of Timezyme Diversification in Vertebrates

**DOI:** 10.1371/journal.pone.0112380

**Published:** 2014-12-08

**Authors:** Damien Cazaméa-Catalan, Laurence Besseau, Jack Falcón, Elodie Magnanou

**Affiliations:** 1 Sorbonne Universités, UPMC Univ Paris 06, UMR 7232, BIOM Biologie Intégrative des Organismes Marins, Observatoire Océanologique, Banyuls/Mer, France; 2 CNRS, UMR 7232, BIOM Biologie Intégrative des Organismes Marins, Observatoire Océanologique, Banyuls/Mer, France; Tel Aviv University, Israel

## Abstract

All biological functions in vertebrates are synchronized with daily and seasonal changes in the environment by the time keeping hormone melatonin. Its nocturnal surge is primarily due to the rhythmic activity of the arylalkylamine *N*-acetyl transferase AANAT, which thus became the focus of many investigations regarding its evolution and function. Various vertebrate isoforms have been reported from cartilaginous fish to mammals but their origin has not been clearly established. Using phylogeny and synteny, we took advantage of the increasing number of available genomes in order to test whether the various rounds of vertebrate whole genome duplications were responsible for the diversification of AANAT. We highlight a gene secondary loss of the AANAT2 in the Sarcopterygii, revealing for the first time that the AAANAT1/2 duplication occurred before the divergence between Actinopterygii (bony fish) and Sarcopterygii (tetrapods, lobe-finned fish, and lungfish). We hypothesize the teleost-specific whole genome duplication (WDG) generated the appearance of the AANAT1a/1b and the AANAT2/2′paralogs, the 2′ isoform being rapidly lost in the teleost common ancestor (ray-finned fish). We also demonstrate the secondary loss of the AANAT1a in a Paracantopterygii (Atlantic cod) and of the 1b in some Ostariophysi (zebrafish and cave fish). Salmonids present an even more diverse set of AANATs that may be due to their specific WGD followed by secondary losses. We propose that vertebrate AANAT diversity resulted from 3 rounds of WGD followed by previously uncharacterized secondary losses. Extant isoforms show subfunctionalized localizations, enzyme activities and affinities that have increased with time since their emergence.

## Introduction

Melatonin is a key hormonal regulator of the biological clock in vertebrates. In all vertebrates investigated so far blood melatonin levels are higher at night than during day and the amplitude of this rhythm varies with season, often reflecting the ambient temperature [1], [2]. Thus the daily and annual variations in the circulating levels of this hormone provide information about the time of day and season, allowing vertebrates to synchronize their metabolism, physiology and behavior to the cyclic variations of the environment [3], [4]. This circulating melatonin is produced by the pineal gland. Melatonin is also synthesized by the vertebrates' retina, where it exerts autocrine/paracrine functions [5]. Melatonin is synthesized from tryptophan in four enzymatic steps. The first is catalyzed by tryptophan hydroxylase (EC 1.14.16.4), producing 5-hydroxytryptophan, which is converted into serotonin by 5-hydroxytryptophan decarboxylase (EC 4.1.1.28). Serotonin is then acetylated by arylalkylamine *N*-acetyltransferase (AANAT, EC 2.3.1.87) to produce *N*-acetylserotonin. The last step, the methylation of *N*-acetylserotonin, is catalyzed by hydroxyindole-*O*-methyltransferase (HIOMT, EC 2.1.1.4.) and produces melatonin [1], [6]. AANAT is important in this pathway because it drives the rhythm of melatonin production, thus earning its nickname “The Timezyme” [7].

AANAT, also named VT-AANAT (for vertebrate AANAT), appeared in vertebrates following the duplication and divergence of an ancestral gene, NV-AANAT (non-vertebrate AANAT), after the Cephalochordate (amphioxus) – vertebrate split over half a billion years ago [8]. The emergence of VT-AANAT was followed by a dramatic acceleration of evolution that accompanied neo-functionalization after the duplication event. This scenario is consistent with the hypotheses that the advent of VT-AANAT contributed to the evolution of the pineal gland and lateral eyes from a common ancestral photodetector [8]. While NV-AANAT disappeared early in vertebrate evolution, the appearance of VT-AANAT marked a crucial step in the development of photodetection and time-keeping in vertebrates [8], [9].

Pineal melatonin secretion is nocturnal in all vertebrates. This conserved pattern masks a unique and dramatic evolution of the mechanisms that regulate its production, both at cellular and organismal levels - see details below and [10]. At the molecular level the regulation of *aanat* gene expression and AANAT enzyme activity are mediated through different pathways of light perception and transmission in tetrapods *vs*. teleosts (ray-finned fish). In addition, while all vertebrates from Cyclostomata (jawless fish) to tetrapods possess one single AANAT, Actinopterygii possess two, or even three, enzymes. Coon and Klein hypothesized that AANAT1 and AANAT2 duplicates arose at the base of the Actinopterygii (bony fish) clade [11]. Interestingly, while AANAT1 is more related to the AANAT from other vertebrates in terms of sequence, AANAT2 has no equivalent [11], [12]. Moreover, AANAT2 is specifically expressed in the pineal gland, while AANAT1 is more specifically expressed in the retina and some brain areas. Finally, some teleosts also express two AANAT1 genes, AANAT1a and AANAT1b. It has been postulated that the emergence of these two forms arose at the split between Ostariophysi and Protacanthopterygii [11].

To date, our knowledge about the diversification of AANAT in teleosts is based on too few species, allowing no definitive conclusions. Here we used both phylogeny and synteny, taking advantage of the increasing number of available genomes, in order to disentangle the effects of repeated vertebrate WGD on AANAT diversification. We reexamine the current theory about AANAT diversification in vertebrates, paying particular attention to the timing of duplication of each AANAT isoform. We show new evidence for various gene secondary losses, giving a more precise scenario not only brings more insight into the evolutionary history of a key vertebrate enzyme, but also allows a better understanding of the crucial steps that may have driven the diversification of the melatonin signaling and synthesis pathways as a whole.

## Material and Methods

### Data Collection

NCBI, Ensembl and genomic databases were screened for publically available AANAT isoforms. Our search was exhaustive for Actinopterygians, but we selected only a subsample of the available AANATs, representative of Sarcopterygii taxa diversity. Cephalochordates, Chondrichthyes and Cyclostomata were also included. Sequences were manually assembled and reannotated whenever required, based on homology with known AANATs.

### Assessments of Conserved Synteny

To examine patterns of conserved synteny, we annotated six genes found upstream and downstream of each aanat paralog in the stickleback genome (*Gasterosteus aculeatus*), used as a reference. Their synteny was compared to all other considered species; *i.e*. eight teleosts: the tetraodon (*Tetraodon nigroviridis*), medaka (*Oryzias latipes*), platyfish (*Xiphophorus maculatus*), tilapia (*Oreochromis niloticus*), Atlantic cod (*Gadus morhua*), zebrafish (*Danio rerio*), and Atlantic salmon (*Salmo salar*). We broaden this approach to one non-teleost Actinopterygii the spotted gar (*Lepisosteus oculatus*) and to four Sarcopterygii including the coelacanth (*Latimeria chalumnae*) and three tetrapods: the Western clawed-frog (*Xenopus tropicalis*), chicken (*Gallus gallus*) and human (*Homo sapiens*). Initial ortholog predictions were visualized using Genomicus v75.01, using its web page interface [13]. To identify additional unannotated genes lying upstream and downstream of the annotated AANAT genes, we also used the program Genscan [14]. The unannotated genes were compared with the non-redundant protein database using the Basic Local Alignment Search Tool (BLAST) [15].

The Atlantic salmon genome was visualized in Genomicus. The salmon genomic data (AGKD00000000.1 [16] was loaded from NCBI (http://www.ncbi.nlm.nih.gov/Traces/wgs/?val=AGKD01) into Geneious Pro 5.4.6 [17], using the Add Sequence Database tool. Contigs containing AANAT isoforms were searched with the Sequence search Tool. Surrounding genes were identified using geneScan and Blast as described previously.

We also screened the genome of the species of interest in search of putative paralogs of AANATs neighboring genes.

To facilitate comparisons, we report the order of genes in the same orientation as they appear in the Stickleback genome assembly, regardless of how they are found in the Ensembl database.

### Sequence Alignment

Amino-acid sequences of known or predicted AANATs were aligned using ClustalW [18] displayed in MEGA 5 [19], and manually adjusted. The alignment was then imported in Geneious Pro 5.4.6 [17].

### Phylogenetic Analyses

Before characterizing the genomic organization of the AANAT genes in nine Actinopterygii (eight teleosts and the spotted gar) and four Sarcopterygii, we performed phylogenetic analyses to reconstruct the duplicative history of the AANAT various isoforms. For this analysis, we added all available Actinopterygii AANAT genes and included sequences from representative of the tetrapods and Chondrichthyes clades for comparisons. We extended our analysis to NV-AANAT forms from Chondrichthyes and Cephalochordates.

Some AANAT isoforms, especially the VT and NV subfamilies, present a high degree of divergence for some regions between investigated species. Thus we selected conserved blocks of the alignment using Gblocks [20], giving a matrix of 167 amino acids. The amino acid alignment was analyzed by maximum likelihood (ML). The model of amino acid substitution was selected in Prottest webserver [21]. The best model based on the Akaike Information Criterion (AIC) was JTT+G (alpha  =  0.76) and was implemented in PhyML displayed in Geneious for further ML reconstructions [22]. Node support was assessed with the bootstrap technique, using 1,000 replicates. Bayesian phylogenetic analyses were also performed on this amino acid matrix using MrBayes 3.0 [23] implemented in Geneious. The Bayesian analysis was performed with the Metropolis-coupled Markov chain Monte Carlo algorithm using a Mixed Model. The tree-space was explored by using four chains run during 4 million generations and saving every 100th tree. The first 25,000 trees were discarded (Burnin') based on preliminary runs that allowed identification of the point when the chains became stationary. A consensus was built with the remaining trees. Bayesian probabilities were used to evaluate branch support.

## Results

### AANAT Isoforms in vertebrates

A total of 90 sequences were selected for this study, 67 isoforms were found for Actinopterygii, 14 were selected to represent Sarcopterygii taxonomic diversity, 8 for Chondrichthyes and Cyclostomata, and one for Cephalochordates. Sequence sources and their annotations are provided in Table 1.

**Table 1:** Sequences accession number for the different AANAT isoforms.

Species	AANAT	Accession number		Remarks/source
		Amino acid sequence	Nucloetide sequence	
*Acipenser sturio*	1	AAY78956.1	DQ077733.1	NCBI
	2	AAY78957.1	DQ077734.1	Partial/NCBI
*Anguilla anguilla*	1	scaffold 6643	Anguilla_anguilla_genome_v1_09_nov_10	Partial/www.zfgenomics.com
	2	scaffold639	Anguilla_anguilla_genome_v1_09_nov_10	
*Anolis carolinensis*	1	ENSACAP00000008194	ENSACAT00000008370	Ensembl
*Arctogadus glacialis*	2	AFQ98400.1	JQ585748	NCBI
*Astyanax mexicanus*	1	ENSAMXT00000016190	ENSAMXG00000015728	Partial/Ensemble
	2	ENSAMXT00000016988	ENSAMXG00000016495	Ensemble
*Bos taurus*	1	NP_803475.1	NM_177509.2	NCBI
*Branchiostoma floridae*	NV beta	ACB14299.1	EU380676.1	NCBI
*Canis lupus familiaris*	1	NP_001070905.1	NM_001077437.1	NCBI
*Callorhinchus milii*	NV	AGX26123.1	KF246476.1	NCBI
	VT	AGX26122.1	KF246475.1	NCBI
*Carassius auratus*	2	ACZ65313.1	AB167078.1	NCBI
*Cyprinus carpio*	1	scaffold8139.1	carp_sept_scaffolds_v3.NEWALL.scaffolds	www.zfgenomics.com
	2	scaffold6738.1	carp_sept_scaffolds_v3.NEWALL.scaffolds	
*Chrysiptera cyanea*	1a	BAN63805.1	AB839763.1	NCBI
	1b	BAN63806.1	AB839764.1	NCBI
*Danio rerio*	1	AAQ54582.1	AY349158.1	NCBI
	2	NP_571486.1	NM_131411.1	NCBI
*Dicentrarchus labrax*	2			Besseau et al., Unpublished data
	1a	ACB13284.1	EU378922	NCBI
	1b	ACB13285.1	EU378923	NCBI
*Esox lucius*	1	AAD21316.1	AF034081.1	NCBI
	2	AAD21317.1	AF034082	NCBI
*Gadus morhua*	2	ENSGMOP00000004524	ENSGMOT00000004660	Ensembl
	1	ENSGMOP00000011553	ENSGMOT00000011867	Ensembl
*Gallus gallus*	1	NP_990489.1	NM_205158.1	NCBI
*Gasterosteus aculeatus*	2	ENSGACP00000018428	ENSGACT00000018464	Ensembl
	1a	ENSGACP00000025480	ENSGACT00000025530	Ensembl
	1b	ENSGACP00000009361	ENSGACT00000009381	Ensembl
*Homo sapiens*	1	NP_001160051.1	NM_001166579.1	NCBI
*Lampetra fluviatilis*	VT	AHA51209.1	KF290562.1	NCBI/partial
*Latimeria chalumnae*	1	ENSLACP00000017693	ENSLACT00000017823	Ensembl
*Lepisosteus oculatus*	1	ENSGACP00000025480_1	ENSGACP00000025480_1	Partial
	2	ENSGACP00000018428_1	ENSGACP00000018428_1	Ensembl
*Lethenteron camtschaticum*	VT		unplaced genomic scaffold scaffold00451	NCBI
*Leucoraja erinacea*	NV	Contig1750401		skatebase.org
	VT	Contig244146, Contig28819 and Contig3043531+ LittleSkate_Transcriptome Contig50898		Partial/skatebase.org
*Macaca mulatta*	1	NP_001040592.1	NM_001047127.1	NCBI
*Mus musculus*	1	NP_033721.1	NM_009591.2	NCBI
*Oncorhynchus keta*	2	AAR07969.1	AY356363.1	Partial/NCBI
	1 brain	AAR07968.1	AY356362.1	NCBI
	1 retina	AAR07967.1	AY356361.1	NCBI
*Oncorhynchus mykiss*	2	AAD25333.1	AF106006.1	NCBI
	1 retina	BAA34809.1	AB007294.1	NCBI
*Oreochromis niloticus*	2	ENSONIP00000001811	ENSONIT00000001810	Ensembl
	1a	ENSONIP00000022987	ENSONIT00000023007	Ensembl
	1b	ENSONIP00000010440	ENSONIT00000010449	Ensembl
*Oryzias latipes*	2	NP_001098316.1	NM_001104846.1	NCBI
	1a	NP_001098302.1	NM_001104832.1	NCBI
	1b	NP_001098330.1	NM_001104860.1	NCBI
*Ovis aries*	1	NP_001009461.1	NM_001009461.1	NCBI
*Oxydoras sifontesi*	2	AFR32910.1	JQ478412.1	NCBI
*Pan troglodytes*	1	NP_001012442.1	NM_001012440.1	NCBI
*Paralichthys olivaceus*	2	AEK70413.1	HQ883478.1	NCBI
*Petromyzon marinus*	VT	AGX26120.1	KF246473.1	NCBI
*Psammomys obesus*	1	AAY79348.1	DQ018371.1	NCBI
*Rana perezi*	1	/	/	Isorna et al., 2006
*Rattus norvegicus*	1	NP_036950.1	NM_012818.2	NCBI
*Salmo salar*	2	contig 354367743	AGKD01	http://www.ncbi.nlm.nih.gov/Traces/wgs/?val=AGKD01
	2	contig 354437952	AGKD01	
	1a	contig 354449422	AGKD01	
	1b	contig 354436144	AGKD01	
	1b	contig 354451409	AGKD01	
*Salvelinus alpinus*	2 VV	/	/	Cazamea-Cataman et al 2013
	2 IA	AFQ98280.1	JQ582623.1	NCBI
*Scophthalmus maximus*	1	ABM21526.1	EF033249.1|	NCBI
	2	ABM21527.1	EF033250.1	NCBI
*Scyliorhinus canicula*	VT	ACB13283.1	EU378921	NCBI
*Siganus guttatus*	1	BAM83582.1	AB781600.1	NCBI
*Solea senegalensis*	2	ACY66881.1	GQ340973.1	NCBI
	1a	ACY66879.1	GQ340971.1	NCBI
	1b	ACY66880.1	GQ340972.1	NCBI
*Sparus aurata*	1	AAT02159.1	AY533402.1	NCBI
	2	AAT02160.2	AY533403.2	NCBI
*Spheroides nephelus*	2	AAL73048.1	AY016023	NCBI
*Takifugu rubripes*	2	ENSTRUP00000029446	ENSTRUT00000029563	Ensembl
	1a	ENSTRUP00000044871	ENSTRUT00000045022	Ensembl
	1b	ENSTRUP00000024942	ENSTRUT00000025045	Ensembl
*Tetraodon nigroviridis*	2	ENSTNIP00000021755	ENSTNIT00000021990	Ensembl
	1a	ENSTNIP00000020660	ENSTNIT00000020893	Ensembl
	1b	ENSTNIP00000006942	ENSTNIT00000007096	Ensembl
*Thynnus thunnus*	2	AFQ98399.1	JQ585747.1	NCBI
*Xenopus laevis*	1a1	AAP57668.1	AY316296.1	NCBI
	1a2	AAP57669.1	AY316297.1	NCBI
*Xenopus tropicalis*	1	ENSXETG00000019534	ENSXETT00000042270	Ensembl
*Xiphophorus maculatus*	2	ENSXMAT00000000129	ENSXMAP0000000012	Ensembl
	1a	ENSXMAP00000006030	ENSXMAT00000006036	Ensembl
	1b	ENSXMAP00000008312	ENSXMAT00000008323	Ensembl

Only sequences used in the phylogenetic and synteny analysis were displayed. Empty cells correspond to sequences obtained in the lab which are under submission to databases. AANATs from genomic databases were manually assembled. The search of Actinopterygii AANATs meant to be exhaustive while we did not include all the publically available tetrapod AANATs. NV and VT stand for the non-vertebrate for and vertebrate AANAT subfamilies of Chondrichthyes and Cyclostomata [8]. Sarcopterygii AANATs were proven to be homologous of the Actinopterygii isoform 1 [4], [11] and were so labeled. Besides these exceptions, isoform description displayed here faithfully reflects the one provided in databases.

**Figure 1:**
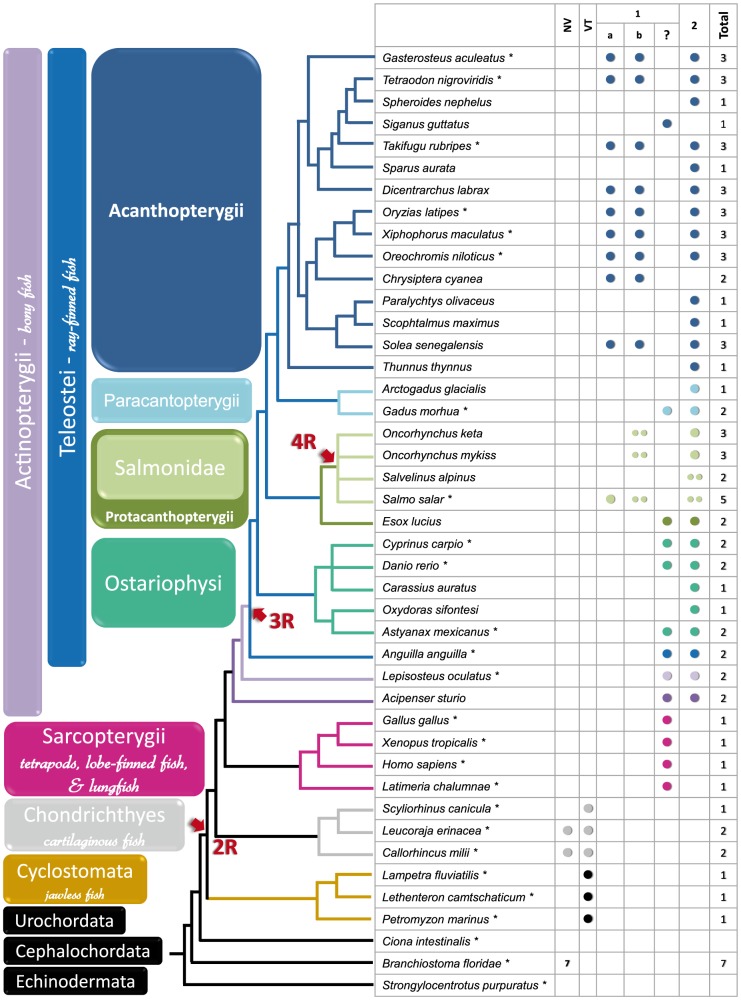
AANAT isoform distribution in vertebrates. AANATs isoforms were sorted according to the nomenclature displayed in table 1. Taxa were classified according to their phylogenetic relationships defined in [65] and available genomes are indicated by an asterisk.

Chondrichthyes are characterized by the presence of two AANAT families: the vertebrate VT- and the NV-AANAT (Fig. 1). Only the VT form was reported in the different lamprey genomes available (Cyclostomata). The amphioxus *Branchiostoma floridae* possesses seven NV-AANATs. The beta form was selected based on its high degree of similarity with Chondrichthyes NV-AANAT, and was used as an outgroup in the phylogeny. Actinopterygii also possess two enzyme subfamilies: the AANAT1 and the AANAT2. Within this clade Acanthopterygii possessed up to three forms: the AANAT1a, AANAT1b and AANAT2. Note that *aanat1b* was not found in any Ostariophysi, including the two species with available genomes (*Danio rerio* and *Cyprinus carpio*). In the same way, no *aanat1a* was ever reported in Paracantopterygii, neither could we locate any *aanat1a* in the *Gadus morhua* genome. Up to five *aanat* genes were identified in the *salmonid Salmo salar* genome including two versions of the *aanat2* and *aanat1b*, and one version of the *aanat1a* gene. Only the AANAT1 has been documented in Sarcopterygii that encompass tetrapods and Actinistia (coelacanth).

### Phylogenetic Relationships among AANAT Genes

**Figure 2:**
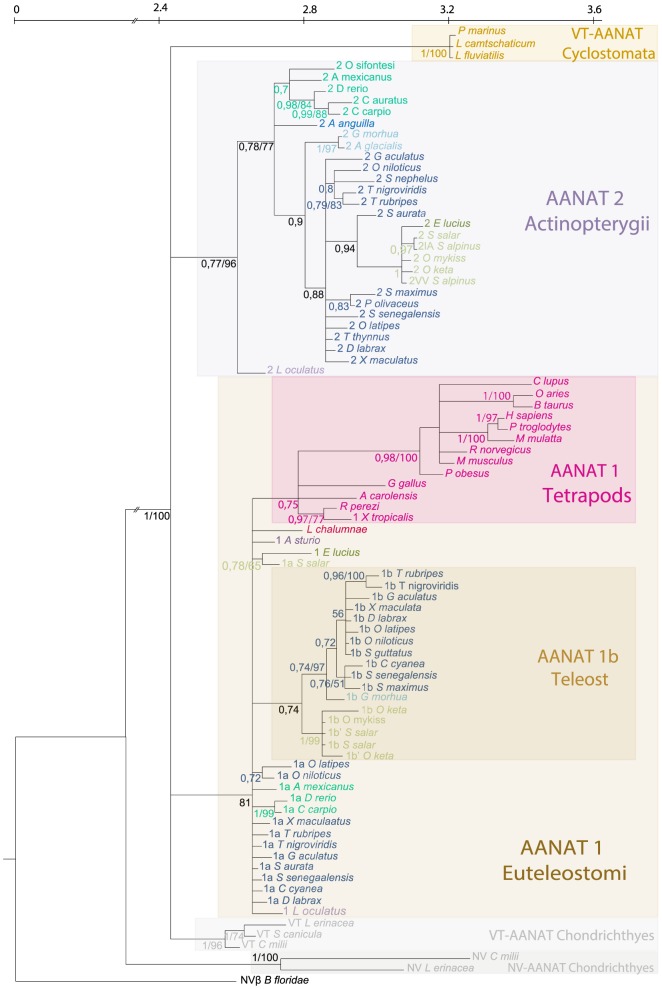
Phylogenetic tree of AANAT isoforms based on amino acid sequences. Bayesian and likelihood methods gave the same tree topology (only the Bayesian tree is shown). The amphioxus *Branchiostoma floridae* NVβ-AANAT was used as outgroup. Numbers on branches indicate from left to right; (a) posterior probabilities in MrBayes reconstructions using a codon model and 4 million generations; (b) bootstrap support obtained in the maximum likelihood (ML) reconstruction (JTT+G) and 1,000 bootstrap replications. Note that posterior probabilities under 0.7 and Bootstrap values <50% were not displayed. Taxa color coding is similar to that used in Figure 1.

Amino acid based Bayesian and ML reconstructions were both performed using the beta NV-AANAT from *B. floridae* as an outgroup. Both techniques gave the same tree topology, showing only very marginal differences, and we chose to feature the Bayesian topology (Fig. 2) including node support provided by the Bayesian and the ML reconstructions (posterior probabilities and bootstrap values respectively). NV- and VT-AANATs are segregated in two robustly supported groups. The overall pattern of VT-AANAT structure shows three well supported clades (posterior probabilities and bootstrap resampling). The first one was constituted of the VT-AANAT form of Chondrichthyes, the second the Actinopterygii AANAT2 form, and the third one gathered all Euteleostomi isoforms 1 (bony vertebrates). Some subgroups are also well supported within the last two clades. AANAT2 grouped by the taxonomic position of the species they belong to: Ostariophysi (*A. mexicanus*; *C. auratus*; *C. carpio*; *D. rerio; O. sifiontesi*); Protacanthopterygii (*E. lucius* and the Salmonidae species); Paracanthopterygii (*G. morhua* and *A. glacialis*); and Pleuronectiforms (*S. maximus*; *P. olivaceus*; *S. senegalensis*). The AANAT1 from Euteleostomi were subdivided into two robust clades including either tetrapod AANATs or teleost AANAT1b, but teleost AANAT1a forms were not robustly grouped.

### Analysis of synteny

**Figure 3:**
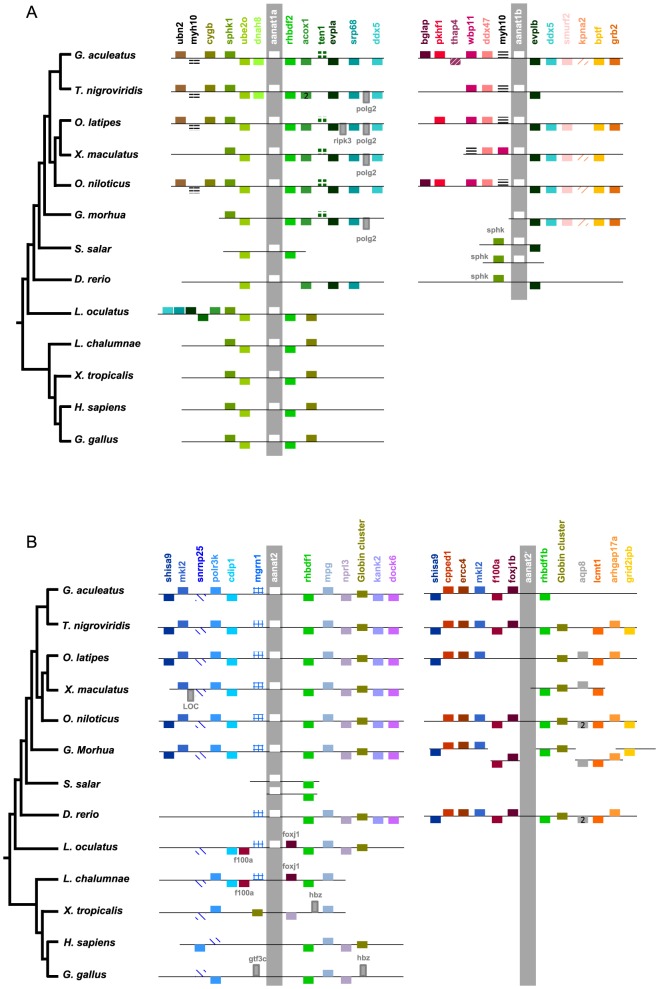
Patterns of synteny in genes neighboring *aanat1* and *aanat2*. Unscaled representations of conserved synteny of *aanat* genes: *aanat1* (A) and *aanat2* (B) paralogons are represented in parallel. The stickleback genome (*Gasterosteus aculeatus*) was used as a reference, and all *aanat* genes are presented in this same orientation. Six genes found upstream and downstream of each *aanat* paralog were selected in the stickleback and sought in all species of interest. Each box represents a gene placed on its chromosome portion. A number two in the box represents rare cases where two consecutive identical genes are found. Genes in the forward orientation are shown on top of the chromosome, whereas genes in the reverse orientation are shown below. The globin cluster encompasses several genes in various orientations, for details see [66]. Precise gene location and name is provided in the S1 Data spreadsheet.

All *aanat* genes were located in a region presenting a noticeable degree of conservation among all of the investigated Actinopterygii. *aanat1* and *aanat2* contigs shared two paralogs: *rhbf2* and *rhbf1*, besides *cygb*, coding for a *cytoglobin*, and the globin cluster that framed *aanat1* and *2* (Fig. 3A-B, and S1 Data for precise gene locations). Four genes (*sphk1*, *ube2o*, *rhbdf2* and *cygb*) were close neighbors of *aanat1a* in Actinopterygii and Sarcopterygii. The Actinopterygii *aanat1b* region found no ortholog in Sarcopterygii.

No *aanat2* gene was found in Sarcopterygii, but eight orthologs of genes neighboring Actinopterygii *aanat2* were encountered (*snrnp25*, *polr3k*, *cdip1*, *mgrn1*, *rhbdf1*, *mpg*, *nprl3* and the globin cluster). The conservation of synteny is particularly strong in the spotted gar (*L. oculatus*) and the coelacanth (*L. chalumnae*), that lie at the base of the Actinopterygii and the Sarcopterygii lineages, respectively. In this region, and apart from the missing *aanat2* in the coelacanth, six genes are conserved between the two species without any insertion/deletion (*cdip1*, *f100a*, *mgrn1*, *foxj1*, *rhbdf1*, *mpg*).

The vast majority of teleosts presented two *aanat1* genes, 1a and 1b, which were part of a paralogon constituted of three genes: *ddx5*, *myh10* and *evpla*/*evplb*. Only *G. morhua* lacked *aanat1a* in the region where all other paralogs of *aanat1b* were present. Conversely, *D. rerio* lacked the *aanat1b* on chromosome 3 where all six paralog genes were present. The same pattern was found in the recently released genome of the blind cave fish *Astyanax mexicanus*: *aanat1b* was absent in its putative location otherwise containing all six paralog genes (data not shown). To investigate whether teleosts also presented two AANAT2 forms, we looked for paralogs of *aanat2* surrounding genes. All teleost genomes (apart from salmonids which we discuss separately below) were characterized by a paralogon of the *aanat2* region composed of *shisa9*, *mkl2*, *f100a*, *rhbdf1* and the globin cluster where the paralog of *aanat2* was missing, and henceforth named aanat2′.

At the time we wrote this article, the *S. salar* available genome was only a primary assembly version with low coverage. We were thus unable to obtain aanat orthologons of the same length as the one displayed for other species. Nevertheless, we found contigs containing the three *aanat* paralogs (1a, 1b, and 2). Two isoforms were identified for the *aanat1b* and *aanat2* genes, whereas only one *aanat1a* gene was encountered. The two *aanat2* nucleotide sequences are almost identical displaying only two synonymous changes. AANAT1b peptide sequences differed by substitution of nine amino-acids a deletion of two amino-acids. Both *aanat1b* were located next to a *sphk* gene whose paralogs were found close to Sarcopterygii and Actinopterygii *aanat1a*.

## Discussion

This study sheds new light on the evolution of AANAT, overturning preconceptions and providing a totally new vision about the diversification of AANATs in the different classes of vertebrates. We have shown previously that both NV- and VT-AANAT enzymes were present in the common ancestor of vertebrates, prior to the split of Cyclostomata and Gnathostomata (jawless and jawed vertebrates) [8]. Rampant gene loss (which rapidly erases signals of genome duplication) and the sparsity of Cyclostomata and Chondrichthyes genomic data precluded investigations on genes neighboring NV- and VT-AANAT. Without a synteny approach, the question of the orthology of Cyclostomata and Chondrichthyes VT-AANAT cannot be solved. Our phylogenetic analysis alone did not reveal whether the VT-AANAT was more closely related to the Euteleostomi. We discuss below new evidence about the time of the AANAT1/AANAT2 and AANAT1a/1b duplication.

### The emergence of two VT-AANAT isoforms

**Figure 4:**
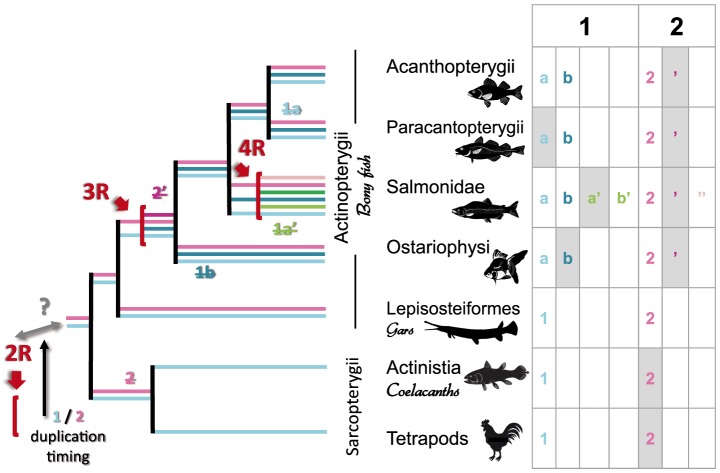
Evolutionary scenario proposing the potential role of whole genome duplications in AANAT diversification. Whole genome duplications are indicated by a red arrow: they concern vertebrates (2R), teleosts (3R) and salmonids (4R). Some *aanat* duplications were followed by secondary losses and are represented by shaded cells in the table or dotted branches on the tree. Other *aanat* paralogs diverged to form various isoforms as shown in different colors when present in a given phylum. The exact timing of the *aanat1*/*2* duplication cannot be inferred from current genomic data and ranges between the 2R and the Actinopterygii/Sarcopterygii split.

The occurrence of the two AANAT1 and AANAT2 forms is specific to all investigated Actinopterygii but is not reported in any Sarcopterygii, namely the Actinistia (coelacanth) and tetrapods (Fig. 1). Until now, the duplication of the ancestral AANAT to form the paralogous products *aanat1* and *aanat2* was assumed to have occurred at the base of the Actinopterygii lineage [11]. Our results contradict this view. Indeed, the present synteny analysis highlights the presence of an orthologon shared by all Euteleostomi (Actinopterygii and Sarcopterygii) in the region where only Sarcopterygii possesses the AANAT2. In other words many genes were conserved both upstream and downstream the region where the *aanat2* gene is now missing, suggesting that the most recent time at which the aanat duplication could have occurred would be in the common ancestor of Actinopterygii and Sarcopterygii, and that the absence of *aanat2* in the coelacanth and tetrapods results from a secondary loss. The loss of this gene probably did not involve deletion of a large genomic portion. The most parsimonious explanation is that the *aanat2* ortholog was lost from the genome by a unique small-scale event in the basal lineage of Sarcopterygii (Fig. 4). Similar patterns of secondary losses in Sarcopterygii, such as those in the opsin gene family, are considered to have played a crucial role in adaptive evolution of vertebrates to their light environment [24].

The emergence of *aanat1* and *aanat2* paralogs is difficult to time based on the current public data. This event is not the result of single gene duplication since Sarcopterygii regions surrounding *aanat1* and the ‘absent’ *aanat2* are characterized by another pair of paralogous genes (*rhbdf2* and *rhbdf1* respectively). It thus at least involved a regional duplication. Our analysis is restricted to the close neighborhood of *aanat* genes and does not allow us to conclude whether this duplication was part of the whole genome duplication (2R) reported at the basis of the vertebrates [25], [26] or if it rather occurred at the basis of Euteleostomi. We favor the first hypothesis as it involves the smallest number of evolutionary events (duplications and losses), while an independent duplication would lead to a less parsimonious scenario.

### AANAT1 and AANAT2 diversification and losses in teleosts

The AANAT1a and AANAT1b forms have only been reported in teleosts. These two paralogs are framed by paralogons in the eight teleost genomes investigated, suggesting that the duplication event leading to these two forms is at least regional. The teleost specific WGD (the 3R or TGD, Teleost Genome Duplication [27]–[32]) may be at the origin of the *aanat1* duplication into the *1a* and *1b* paralogs, and remains the most parsimonious scenario. This hypothesis tacitly implies that *aanat2* was duplicated in the same event. Yet no *aanat2* paralogs were found when screening the available teleost genomes. However paralogs were found for four of the genes framing *aanat2*, constituting a paralogon of the *aanat2* region (Fig. 3B). This conservation of synteny observed in all investigated teleosts suggests a secondary loss of *aanat2*′, the *aanat2* paralog in their common ancestor. These eight species belong to a wide diversity of taxa (Acantopterigii, Paracantopterygii, Protacanthopterygii, and Ostariophysi), suggesting that the secondary loss of aanat2′ occurred shortly after the TGD, in the common ancestor of those taxa, *i.e*., before the diversification of teleosts. Our findings change the prevailing scenario [11] that was built using fewer aanat1a and 1b available sequences. Coon and Klein (2006) assumed that the duplication occurred in the main trunk of the teleost linage after the zebrafish branch had split off, and did not consider the secondary loss of a putative second AANAT2 form.

Up to now, the evolutionary history of teleosts AANAT1a and AANAT1b has not led to any straightforward conclusion given that their pattern of presence/absence does not seem to be clade specific. Moreover, the investigation effort exerted to characterize AANAT enzymes has not been the same in all species and for all isoforms, and the well-known aphorism prevails here: “absence of evidence is not evidence of absence”. Our study brings some clarification.

First, the phylogeny analysis allows the AANAT1 nomenclature to be revised, especially for species where only one single AANAT1 form is known. Based on its position in the AANAT phylogeny, the orange-spotted spinefoot *Siganus guttatus* AANAT1 can be identified as a 1a form. All known Protacanthopterygii AANAT1 may all be the 1b form: the pike *E. lucius*, the rainbow trout *O. mykiss*, and the two undefined AANAT1 forms of chum salmon *O. keta* belong to the well supported 1b clade. But the phylogeny alone does not solve the question of AANAT1 nomenclature in the blind cave fish (*Astyanax mexicanus*), the common carp (*Cyprinus carpio* and the zebrafish (*Danio rerio*) for which sequences fall in an intermediate position between 1a and 1b (Fig. 2). The synteny analysis allows this question to be circumvented, since this *aanat1* is located in the ortholog region of all other teleosts *aanat1a*. In addition, *aanat1b* is clearly absent in these three species whose genomes are available. Moreover, all genes surrounding *aanat1b* in other teleosts present a conservation of synteny, demonstrating the secondary loss of *aanat1b* in the blind cave fish *A. mexicanus*, the common carp *C. carpio* and the zebrafish *D. rerio*. Further investigations would be needed to generalize this statement for all Ostariophysi, but we hypothesize here that this event may have happened in the common ancestor of this clade (Fig. 4). Other genomic approaches on HOX genes have already shown that *D. rerio* presents a unique pattern of gene duplication/loss compared to other teleosts [33].

Only one *aanat1* was detected in the Protacanthopterygii G. *morhua* genome. This Atlantic cod form is closely related to other teleost *aanat1b* (Fig. 2). Its neighboring genes correspond to the 1b paralogon, while the 1a is missing in the region where its ortholog is found in other teleosts (Fig. 3A). This information allows us to conclude that there was a subsequent loss of *aanat1a* in the Atlantic cod G. *morhua* (Fig. 4). The pattern of presence/absence of AANAT1a/AANAT1b in Ostariophysi and Paracantopterygii results from the reciprocal gene losses (RGL) previously defined [34]. The two paralogs were created by WGD (in this case the TGD) and were retained until speciation, after which each taxon lost a different copy.

### A round of duplications specific to salmonids

The Atlantic salmon *S. salar* presents a unique diversity of AANATs with more than the three typical *aanat1a*, *aanat1b* and *aanat2* forms classically encountered in other teleosts. Its genome contains one form of *aanat1a*, but two forms for both, *aanat1b* and *aanat2*. Although the salmon genome is still poor and does not allow synteny analysis on long fragments, we are confident each duplicate of the *aanat1b* and *aanat2* are located on two distinct genomic regions and truly correspond to two different gene copies (S1 Data). First, each belongs to a distinct contig, also containing respectively the *sphk* or *rhbdf1* genes. Second, these *aanat1b* or *aanat2* paralogons present more genetic divergence than sequencing errors alone could explain. But they are much less divergent than two paralogs generated by the TGD*: rhbdf1/1b* located on a given teleost *aanat2*/*2*′ paralogon show 75 to 80% identity, while the two salmon *rhbdf1* neighboring these two *aanat2* copies show 94% identity.

Other multiple AANAT isoforms were previously reported in related salmonid taxa. Two AANAT1b were observed in the chum salmon *O. keta* [35] and two AANAT2 were characterized in the Arctic char *S. alpinus* [36], [37]. Unlike in *S. alpinus* where the two variants differ by two amino-acids, the *S. salar* AANAT2 variants only differ by two nucleotides and are identical at the peptide level.

A specific WGD [38], [39] which occurred in the Salmonidae family at least 88 Mya [40] could explain this particular AANAT diversity. We hypothesize the three *aanat* genes - resulting from two previous whole genome duplication rounds (2R and the TGD 3R) and a subsequent loss of the *aanat2*′ - could have been duplicated during this event. We propose that the second copy of the *aanat1a* has been lost. Other mechanisms such as independent regional duplications could also explain this pattern. Given this last scenario is less parsimonious we favor the one involving the well documented salmonid WGD [38], [39].

### Functional implications of AANAT diversification

We suggest that *aanat* genes present in vertebrates went through a series of three consecutive duplications. Based on available evidence we propose these duplications are the result of the Vertebrata, teleosts and salmonid-specific WGDs. Several *aanat* duplicates were lost, possibly after an accumulation of deleterious mutations or chromosome rearrangements. This is mainly the case for the most ancient duplicates such as the NV- and VT-*aanat* in early vertebrates. Secondary losses are suspected to be the most frequent scenario following gene duplication, because only a minority of gene pairs adopts a new function (neofunctionalization) or partitions old functions (subfunctionalization) quickly enough to persist in face of deleterious mutations [26], [41], [42].

Falcon and colleagues [8] demonstrated that after an ancestral NV-*aanat* gene duplication in early vertebrates, one copy acquired the new function of melatonin production while the other copy maintained its detoxification role and was eventually lost in most Vertebrate lineages. This scenario obviously corresponds to a neofunctionalization event. Based on tissue or organ localization, enzyme activity and substrate affinity, we see that that all VT-*aanat* duplications leading to the AANAT1/AANAT2 forms, and subsequently the AANAT1a/AANAT1b forms, did not generate new functions but rather led to subfunctionalization or functional refinement or shuffling. These subfunctions are more diverged in the AANAT1 and AANAT2 forms that emerged earlier in the evolutionary history of the Timezyme. On the contrary, finer specializations are seen between the AANAT1a and AANAT1b isoforms, whose duplication and differentiation is more recent.

The acquisition of *aanat1*/*aanat2* paralogs occurred before the divergence of Sarcopterygii and Actinopterygii and we clearly deciphered the two distinct evolutionary histories followed in each lineage. Both genes were maintained in Actinopterygii, possibly corresponding to the complementary partitioning of ancestral structural or regulatory functions between *aanat1* and *aanat2*. Several clues favor this hypothesis. A first one is the selective expression of the paralogs; *aanat1* is expressed in the retina and brain, while *aanat2* is expressed in the pineal gland [43]–[45]. A second one is the partitioning and refinement of the kinetics properties of these enzymes. AANAT1 acetylates indoleethylamines and phenylethylamines equally well, whereas AANAT2 exhibits strong preference for indoleethylamines [43], [46]–[48]. A third one is the difference in regulation by temperature both at the cellular [49], [50] – and molecular levels [43], [46]. The subfunctionalization of AANAT1 and 2 may have contributed to the functional specialization of the organs where they are respectively expressed. In the retina, acetylation of both *N*-acetylserotonin (indoleethylamine) and dopamine (phenylethylamine) contributes to regulating the circadian rhythmicity displayed by the tissue, which involves the reciprocal melatonin/dopamine interaction [51]–[53]. The pineal gland became a melatonin factory that allows integrating the photoperiod and temperature information in a species-dependent manner. This pattern typically corresponds to the duplication, degeneration, complementation (or DDC) model where the addition of both paralogs' functions may provide at least that of the pre-duplicated gene [26].

On the contrary, *aanat2* was lost in Sarcopterygii and only the *aanat1* paralog remained. This is the most frequently observed scenario after duplication [26], [41]. Even if gene redundancy may be maintained for a certain period, nonfunctionalization (or pseudogenization) by accumulation of detrimental mutations is the most common fate of one of the duplicates. However, this loss of *aanat2* does not mean a loss of function. The remaining Sarcopterygii AANAT gathers functions observed for both Actinopterygii AANAT1 and AANAT2. Phylogeny and synteny demonstrate that this gene is the ortholog of Actinopterygii *aanat1*. Yet, Sarcopterygii expression sites resemble more Actinopterygii AANAT2. Except for frog AANAT, which is equally expressed in the retina, the pineal photoreceptors and in some discrete brain areas [54], chicken and mammalian AANAT display high levels of expression in the pineal gland and much lower levels in the retina [6], [55]. The kinetics of the avian and mammalian AANAT appear to be closer to teleost AANAT2 rather than teleost AANAT1 in terms of substrate selectivity, while displaying an even higher catalytic efficiency [56]–[61]. *Aanat* expression and AANAT enzyme activity are both higher at night than during day, as is always the case for teleost AANAT2 but not necessarily for teleost AANAT1. To summarize, Sarcopterygii AANAT is the ortholog of retinal AANAT1 from Actinopterygii but displays many characteristic of the Actinopterygii AANAT2 pineal enzyme, which controls production of circulating melatonin. The two orthologs (*i.e.* Sarcopterygii AANAT and Actinopterygii AANAT2) either display a convergent evolution towards the endocrine production of melatonin or retained the ancestral features of the AANAT from which they diverged. Finer characterization of early vertebrate AANAT should help decipher the functional features of the ancestral VT-AANAT. In any case the present pattern highlights the crucial role of melatonin in vertebrate biological rhythms.

The physiological refinement produced by the split of AANAT1 into the 1a and 1b forms is more tenuous. While some species possess both genes, others possess either one or the other, as a result of gene secondary losses. When both genes are present *aanat1b* shows higher levels of expression. However this observation concerns only two species, the sole *Solea senegalensis* [62] and the sea bass *Dicentrarchus labrax* (Besseau et al, unpublished data). While we observe a marked specialization in expression sites for AANAT1s (retina/brain) *vs*. AANAT2 (pineal) the subfunctionalization of AANAT1a/1b is only observed at the cell type level. It concerns different cell layers of the retina (Besseau et al, unpublished data) or a different timing in expression across larval development [62]. Information concerning the kinetics and tissue distributions of the two isoforms is lacking. It is possible that expression of either form is related to some specific life history traits of the species considered.

We show that *aanat1b* has been lost by three Ostariophysi (*D. rerio*, *C. carpio*, and *A. mexicanus*). Based on our phylogeny, the primary structure of these AANAT1a is an intermediate between AANAT1a and 1b orthologs from other teleosts. This would provide a case of functional shuffling where surviving paralogs re-acquire subfunctions related to loss of ohnologs (OGMs, Ohnologs Gone Missing) [26]. Further functional assays would be suitable to test the hypothesis that these Ostariophysi AANAT1a enzymes have re-acquired subfunctions lost with the 1b paralog.

In summary vertebrates provide a panel of evolutionary strategies with regard to the synthesis of melatonin, wherein the Timezyme plays a pivotal role. As demonstrated earlier, VT-AANAT results from a duplication followed by neofunctionalization (acetylation of indolamines) of one paralog and disappearance of the other [8]. This new AANAT displayed a more specific tissue distribution than its paralog (as seen when both are present, *i.e*., in Chondrichthyes) [8]. This specialization may be at the origin of the nocturnal melatonin secretion signal. From there, and after a new duplication event, two scenarios are observed. The lack of public information and the incomplete nature of released genomes of the Cyclostomata and Chondrichthyes preclude providing a definitive picture based on synteny. However, our data support the idea that the 2R generated AANAT1 and AANAT2 and that the two paralogs were conserved only in Actinopterygii, with clear subfunctionalization and tissue specific expression. These factors might have been crucial in the conservation of AANAT2 by Actinopterygii. From Cyclostomata to mammals AANAT expression became concentrated in the pineal gland and catalytic efficiency increased. Thus, whatever the strategy, they all converged to make pineal melatonin production the signal for the night from Cyclostomata to mammals. In other words, the time-keeping nocturnal signal is preserved. This common outcome observed at the molecular level has been accompanied by spectacular modifications of the pineal anatomy and structure as well as of the mechanisms of regulation by light, as extensively reported elsewhere [10],[63],[64], emphasizing the importance of the melatonin message in vertebrates.

## Supporting Information

S1 Data **Detailed information about genes involved in the synteny analysis.** Each of the four tables displays precise name, accession number and Ensembl location of genes involved in the synteny analysis for aanat1a, aanat1b, aanat2, and aanat2′. Six genes found upstream and downstream of each aanat paralog were annotated, using the stickleback genome (*Gasterosteus aculeatus*) as a reference. Cells highlighted in yellow provide the contig number on which were found the targeted genes in the AGKD01 salmon genome, since *S. salar* is not referenced in Ensembl.
(XLSX)
